# The impact of multidisciplinary team nutrition management on nutritional and toxicity status in patients with nasopharyngeal carcinoma

**DOI:** 10.1016/j.apjon.2023.100237

**Published:** 2023-04-16

**Authors:** Xueqing Ou, Hui Chen, Ting Qiu, Yajun Yuan, Xiaohua Gong

**Affiliations:** a Department of Cancer Center, The Fifth Affiliated Hospital of Sun Yat-sen University, Zhuhai, China; b Guangdong Provincial Key Laboratory of Biomedical Imaging, The Fifth Affiliated Hospital of Sun Yat-sen University, Zhuhai, China

**Keywords:** Multidisciplinary, Nutrition, Chemoradiotherapy, Nasopharyngeal carcinoma, Toxicity

## Abstract

**Objective:**

To explore the impact of multidisciplinary team (MDT) nutrition management on the nutritional and toxicity status of patients with nasopharyngeal carcinoma undergoing chemoradiotherapy.

**Methods:**

A total of 104 patients undergoing chemoradiotherapy for nasopharyngeal carcinoma admitted to our hospital from July 2018 to February 2021 were retrospectively enrolled, including who received conventional nutrition management (the routine group, *n* ​= ​52) and who received MDT nutrition management (the experimental group, *n* ​= ​52). Nutritional indicators (dietary intake, body mass index, serum albumin, serum prealbumin, hemoglobin, total lymphocyte count, serum transferrin [TRF]), the Nutrition Risk Screening 2002 (NRS2002) score and acute toxicity level were recorded before, during, and after chemoradiotherapy. Multiple regression analysis was performed to identify nutritional risk indicators.

**Results:**

During and after chemoradiotherapy, the body mass index, albumin, prealbumin, hemoglobin, total lymphocyte count, TRF, dietary intake, number of patients with an NRS2002 score < 3, and acute toxicity score in the experimental group improved compared to those in the routine group (*P* ​< ​0.05). Concurrent chemotherapy, the NRS2002 score and a half-diet strategy were independent factors affecting the nutritional status of nasopharyngeal carcinoma patients who underwent chemoradiotherapy.

**Conclusions:**

Active screening and evaluation of the nutritional status of patients with nasopharyngeal carcinoma during chemoradiotherapy as well as MDT nutrition management can be used to detect nutritional problems, thus improving quality of life and reducing related toxicity.

## Introduction

Cancer is one of the major chronic diseases affecting the health of the global population. Nasopharyngeal carcinoma (NPC) is a kind of head and neck cancer, with complex and diverse causes and high incidence rate in South China.^1^^,^^2^ Chemoradiotherapy (CRT) is the key to disease control and increasing the survival rate. The local control rate in early NPC patients is 70%–90%.^1^^,^^2^ However, CRT often causes a variety of side effects, including oral mucosa and skin damage, anorexia and anemia, leading to malnutrition. Several studies have indicated that patients with NPC undergoing CRT have the highest incidence of malnutrition.^2^, ^3^, ^4^ Long-term nutritional deficiencies increase the chance of infection and damage multiple organ functions, resulting in prolonged recovery, which also reduces the overall survival rate and distant metastasis-free survival rate.^5^, ^6^, ^7^ Patient-centered, multidisciplinary teams conduct clinical discussions on a particular disease and systematically formulate a standardized, individualized, and continuous comprehensive treatment plan, which is implemented by related disciplines individually or jointly with efficacy, improving diagnostic and treatment ability. In European countries and the US, multidisciplinary teams have become the most vital model for saving patients having cancer, promoting disease recovery and significantly improving the survival rate.^8^^,^^9^ Multidisciplinary team professional committees that regulate the multidisciplinary team model on diagnosis and treatment of NPC were established in China.^10^ Moreover, many tertiary-grade class-A hospitals have extensively implemented the multidisciplinary team model, which has achieved remarkable success in the diagnosis of intractable diseases, providing the best treatment plan for patients with a clear diagnosis and addressing the follow-up difficulties caused by chronic diseases. However, there are still some urgent problems to be solved by multidisciplinary teams. First, the composition and the structural characteristics of a multidisciplinary team can lead to the uneven distribution of benefits as well as insufficient organizational incentives and risk-sharing among members. Second, it is challenging to control the application scope of the multidisciplinary team and improve the service efficiency of high-quality resources. Multidisciplinary teams have not been fully developed in China, and a large amount of research data is required to provide clinical references. Therefore, this study aims to explore the effects of multidisciplinary team nutrition management on the nutritional status and incidence of toxicity in patients undergoing CRT for nasopharyngeal cancer and confirms the methods and main points of multidisciplinary teams to provide a new strategic basis for clinical application.

## Methods

### Study design and participants

Patients undergoing CRT for nasopharyngeal cancer in our hospital from July 2018 to February 2021 were retrospectively enrolled. All patients were informed of this study and voluntarily signed a consent form for admission. The inclusion criteria were a medical history, symptoms, and pathological examination indicating NPC; indications for chemotherapy and radical radiotherapy for the first time in the hospital; an age of 18–70 years; an educational level above the elementary school level, with certain reading and communication skills; no other serious primary malignant tumors; the availability of data on nutritional risk according to the European Nutrition Risk Screening 2002 (NRS2002) assessment form upon admission^11^; healthy liver, kidney, and heart functions; and no major organic diseases. The exclusion criteria were an estimated survival time < 3 months, illiteracy, poor basic vital signs, a history of taking drugs that affect body metabolism in the month prior to the operation, and combined mental illness or a severe communication disorder. Those with incomplete case data, imperfect examinations and those who died or were lost to follow-up were also excluded. The allocation of MDT or routine nutrition management was under consideration of individual doctor.

### Measure

#### Treatment

According to the relevant diagnosis and treatment consensus, 10 patients were irradiated with a 6-MV photon beam using an infinity linear accelerator (ELEKTA, Stockholm, Sweden) for image-guided radiation therapy. The patients in stage II received concurrent radiotherapy and chemotherapy (cisplatin 80–100 mg/m^2^, day 1, every 3 weeks), and those in stage III–IV a received two cycles of induction chemotherapy followed by concurrent radiotherapy. Induction chemotherapy was performed using the Cisplatin+5-fluorouracil (PF), Gemcitabine+cisplatin (GP), or Paclitaxel+cisplatin (TP) regimen. For radiotherapy, intensity-modulated radiotherapy was mandatory in this trial. The clinical target volume was delineated as the gross tumor volume plus an additional 5–10 ​mm margin. The prescribed doses were 60–70 ​Gy in 27–35 fractions (2.00–2.36 ​Gy per fraction) for the planning target volumes derived from the gross tumor volume of the primary site and of the lymph nodes, and 50–60 ​Gy for that derived from the clinical target volume, with five daily fractions per week.

#### Nutrition management

Nutrition management during CRT in the routine group comprised symptomatic treatment; general nursing and observations of condition; discharge health education; an individualized nutrition management plan based on the actual clinical situation, including dietary guidance and health education; adjustment of nutrient solution components; supplementation with high-calorie, high-protein foods, such as concentrated sugar, protein, and fat; and oral nutritional supplements or a short-term nasal feeding tube for patients who had difficulties with oral three-step analgesics. Conventional nursing and a multidisciplinary team were employed for the experimental group. For preliminary preparation, the multidisciplinary team included senior doctors from the departments of head and neck oncology, otorhinolaryngology, psychiatry, and rehabilitation and nutrition who recorded clinical data, focused on nasopharyngeal tumor diseases to collect, analyze, discuss, and communicate the patients' physiological, psychological, and social problems, and formulated a proper diagnostic and treatment plan based on the patients’ physiological and psychological characteristics. For plan implementation, clinicians from the departments of head and neck oncology and otorhinolaryngology chose the definitive therapy, provided medication guidance for diseases and related complications, provided relevant health education and performed preventive work. The department of rehabilitation monitored nerve functions and comprehensively treated concurrent multiple organ and tissue damage. The departments of psychiatry and nutrition assessed physical condition and psychological status to develop appropriate exercise and diet plans, integrated case data from various medical doctors and conducted comprehensive nutrition management. In addition to conventional nutrition management, tumor, neurological, and endocrine function features were considered. The physicians from all departments maintained lines of communication and tackled various problems to correct and improve the diagnosis and treatment. Each patient would spend a total of about 2 ​h every week to receive an evaluation in each specialty of the MDT group, and then the case management nurse would conduct a comprehensive evaluation of the patient data, and then feedback to each doctor and nurse participating in the group, and then adjusted the treatment of the patient. The biggest feature of nutritional intervention in the experimental group was that there was a multidisciplinary team involved in patient care, and patients can receive comprehensive nutritional and psychological support.

#### Observation indicators

The nutritional indicators of the two groups of patients were recorded before, during (week 4 of radiotherapy), and after radiotherapy (at the end of radiotherapy), including the body mass index (BMI), serum albumin (ALB), serum prealbumin (PAB), hemoglobin (Hb), total lymphocyte count (TLC), and serum transferrin (TRF). The detection reagents were standard reagents. The NRS2002 score and acute reflex response score were recorded before, during (week 4 of radiotherapy), and after CRT (at the end of CRT).

### Data analysis

SPSS 22.0 software (SPSS Inc., Chicago, IL, USA) was used for statistical analysis. The paired *t*-test and chi-square test were selected based on the variable type and type of comparison desired. A *P* value < 0.05 was considered to indicate significance.

### Ethical considerations

This study was reviewed and approved by the Ethics Committee of the Fifth Affiliated Hospital of Sun Yat-sen University (IRB No. K240-1). All participants gave signed informed consent to participate.

## Results

### Patient characteristics

A total of 104 patients who strictly met the inclusion criteria were randomly divided into the routine group (conventional nutrition management, *n* ​= ​52) and the experimental group (multidisciplinary team nutrition management, *n* ​= ​52), and all patients had undifferentiated carcinoma. In the routine group, there were 29 males and 23 females, who were 34–66 years old (51.96 ​± ​6.33 years). There were 9 cases with a BMI < 18.5 ​kg/m^2^, 14 cases with stage II disease, 28 cases with stage III disease and 10 cases with stage IV a disease. In the experimental group, there were 31 males and 21 females, who were 32–68 years old (50.21 ​± ​6.50 years). There were 11 cases with a BMI < 18.5 ​kg/m^2^, 15 cases with stage II disease, 29 cases with stage III disease, and 8 cases with stage IVa disease. No significant differences were observed in the general data between the two groups (*P* ​> ​0.05; Table 1). 32 patients in the routine group and 26 patients in the experimental group received nutrition interventions (*P* ​> ​0.05), such as intravenous nutrition support.

**Table 1:** Patients characteristics (*N* ​= ​104).

Characteristics	Experimental group (*n* = 52), *n* (%)	Routine group (*n* = 52), *n* (%)
Age (years)		
Range	32–68	34–66
Median	50.20	51.90
Gender		
Male	31 (59.61)	29 (55.76)
Female	21 (40.38)	23 (44.23)
Stage		
II	15 (28.85)	14 (26.92)
III	29 (55.77)	28 (53.85)
IVa	8 (15.38)	10 (19.23)
BMI < 18.5 ​kg/m^2^	11 (21.15)	9 (17.31)

BMI, body mass index.

### Differences in nutritional indicators between the experimental group and the routine group

No significant differences in BMI, ALB, PAB, Hb, TLC, or TRF were observed between the two groups before CRT (*P* ​> ​0.05). The BMI, ALB, PAB, Hb, TLC, and TRF in both groups were lower after CRT than before CRT (*P* ​< ​0.05). The BMI, ALB, PAB, Hb, TLC, and TRF in the experimental group were higher during and after CRT than those in the routine group (*P* ​< ​0.05; Table 2).

**Table 2:** Comparison of nutritional indicators between experimental group and routine group (*N* ​= ​104, Mean ​± ​SD).

Indicators	Experimental group (*n* = 52)			Routine group (*n* = 52)		
	Before CRT	During CRT	After CRT	Before CRT	During CRT	After CRT
BMI (kg/m^2^)	22.26 ​± ​2.68	21.77 ​± ​2.40^b^	20.48 ​± ​2.33^ac^	22.30 ​± ​2.66	20.56 ​± ​2.27	19.38 ​± ​2.14^a^
ALB (g/L)	44.22 ​± ​4.05	43.66 ​± ​3.87^b^	42.79 ​± ​3.65^ac^	44.18 ​± ​3.98	42.09 ​± ​3.68	40.14 ​± ​3.44^a^
PAB (mg/L)	258.85 ​± ​68.78	239.97 ​± ​52.74^b^	221.54 ​± ​49.96^ac^	260.10 ​± ​69.01	224.46 ​± ​50.05	209.77 ​± ​42.16^a^
Hb (g/L)	129.63 ​± ​11.36	122.22 ​± ​10.09^b^	119.87 ​± ​9.64^ac^	128.87 ​± ​11.22	111.40 ​± ​9.63	92.02 ​± ​8.84^a^
TLC (×10^9^/L)	1.39 ​± ​0.55	1.22 ​± ​0.39^b^	1.08 ​± ​0.22^ac^	1.36 ​± ​0.50	1.09 ​± ​0.33	0.86 ​± ​0.17^a^
TRF (g/L)	2.55 ​± ​0.63	2.46 ​± ​0.55^b^	2.39 ​± ​0.48^ac^	2.57 ​± ​0.65	2.30 ​± ​0.47	2.21 ​± ​0.32^a^

ALB, albumin; BMI, body mass index; CRT, chemoradiotherapy; Hb, hemoglobin; PAB, prealbumin; TLC, total lymphocyte count; TRF, transferrin.

^a^*P* ​< ​0.05 compared with before CRT; inter-group comparison.

^b^*P* ​< ​0.05 compared with routine group during CRT.

^c^*P* ​< ​0.05 compared with routine group after CRT.

### Dietary intake

No significant difference in dietary intake was observed between the two groups before CRT (*P* ​> ​0.05). More cases of dietary reduction ≥ 1/2 were observed after CRT in the two groups than before CRT. More cases of dietary reduction < 1/2 were observed in the experimental group during and after CRT than in the routine group (*P* ​< ​0.05), more cases of dietary reduction ≥ 1/2 were observed in the routine group during and after CRT than in the experimental group (*P* < 0.05; Table 3).

**Table 3:** Comparison of dietary intake between experimental group and routine group (*N* ​= ​104).

Dietary intake	Experimental group (*n* = 52), *n* (%)			Routine group (*n* = 52), *n* (%)		
	Before CRT	During CRT	After CRT	Before CRT	During CRT	After CRT
Dietary reduction ≥ 1/2	6 (11.54)	17 (32.69)^b^	22 (42.31)^ac^	5 (9.62)	23 (44.23)	36 (69.23)^a^
Dietary reduction < 1/2	46 (88.46)	35 (67.31)^b^	30 (57.69)^ac^	47 (90.38)	29 (55.77)	16 (30.77)

CRT, chemoradiotherapy.

^a^*P* ​< ​0.05 compared with before CRT; inter-group comparison.

^b^*P* ​< ​0.05 compared with routine group during CRT.

^c^*P* ​< ​0.05 compared with routine group after CRT.

### NRS2002 score

No significant difference in the NRS2002 score was observed between the groups before CRT (*P* ​> ​0.05). Fewer cases of an NRS2002 score < 3 were observed in both groups after CRT than before CRT. More cases of NRS2002 score < 3 were observed in the experimental group during and after CRT than in the routine group (*P* ​< ​0.05), more cases of NRS2002 score ≥ 3 were observed in the routine group group during and after CRT than in the experimental (*P* < 0.05; Table 4).

**Table 4:** Comparison of NRS2002 scores between experimental group and routine group (*N* ​= ​104).

NRS2002 scores	Experimental group (*n* = 52), *n* (%)			Routine group (*n* = 52), *n* (%)		
	Before CRT	During CRT	After CRT	Before CRT	During CRT	After CRT
≥ 3 scores	12 (23.08)	18 (34.62)^b^	21 (40.38)^ac^	11 (21.15)	26 (50.00)	34 (65.38)
< 3 scores	40 (76.92)	34 (65.38)^b^	31 (59.62)^a^^c^	41 (78.85)	26 (50.00)	18 (34.62)^a^

CRT, chemoradiotherapy.

^a^*P* ​< ​0.05 compared with before CRT; inter-group comparison.

^b^*P* ​< ​0.05 compared with routine group during CRT.

^c^*P* ​< ​0.05 compared with routine group after CRT.

### Acute toxicity

The acute toxicity level in both groups was higher after CRT than during CRT (*P* ​< ​0.05). The acute toxicity level in the experimental group was lower than that in the routine group during and after CRT (*P* ​< ​0.05; Table 5).

**Table 5:** Comparison of acute toxicity between experimental group and routine group (*N* ​= ​104).

Acute toxicity	Experimental group (*n* = 52), Mean ± SD		Routine group (*n* = 52), Mean ± SD	
	During CRT	After CRT	During CRT	After CRT
Neutropenia	1.02 ​± ​0.37^b^	1.30 ​± ​0.55^ac^	1.05 ​± ​0.34	1.77 ​± ​0.63^a^
Cutireaction	1.01 ​± ​0.44^b^	1.78 ​± ​0.52^ac^	1.03 ​± ​0.42	2.62 ​± ​0.68^a^
Mucosa reaction	1.14 ​± ​0.51^b^	1.77 ​± ​0.60^ac^	1.18 ​± ​0.52	2.68 ​± ​0.71^a^
Swallowing function	1.33 ​± ​0.66^b^	1.98 ​± ​0.79^ac^	1.35 ​± ​0.68	2.74 ​± ​0.89^a^
Xerostomia	1.06 ​± ​0.42^b^	1.38 ​± ​0.55^ac^	1.09 ​± ​0.45	2.01 ​± ​0.67^a^
Nausea and vomiting	1.08 ​± ​0.47^b^	1.39 ​± ​0.60^a^	1.12 ​± ​0.50	1.87 ​± ​0.77^a^

CRT, chemoradiotherapy.

^a^*P* ​< ​0.05 compared with before CRT; inter-group comparison.

^b^*P* ​< ​0.05 compared with routine group during CRT.

^c^*P* ​< ​0.05 compared with routine group after CRT.

### Multiple linear regression analysis of nutritional status

Multiple linear regression analysis was conducted with gender, age, BMI, tumor type, tumor stage, ALB, PAB, Hb, TLC, TRF, diet, the NRS2002 score, acute toxicity level, and chemotherapy as independent variables. The stepwise screening method was applied for the regression analysis, and the final factors in the equation that affected nutritional status were age, BMI, PAB, the NRS2002 score, acute toxicity level, diet reduction, and concurrent chemotherapy (*P* ​> ​0.05; Table 6).

**Table 6:** Multiple linear regression equation of nutritional status (*N* ​= ​104).

Independent variable	*β*	*t*	*P*
Constant	36.696	8.969	0.001
BMI	6.897	3.020	0.046
PAB	9.663	2.014	0.035
Dietary reduction	30.007	6.054	0.008
NRS2002 score	29.636	6.665	0.004
Acute toxicity	1.230	2.014	0.039
Concurrent chemoradiotherapy	32.254	6.554	0.001

BMI, body mass index; PAB, prealbumin; NRS2002, Nutrition Risk Screening 2002.

## Discussion

Patients having cancer can experience treatment-related side effects that impact nutrition status, as well as unwanted weight loss and poor dietary quality. They were interested in and providers supportive of integrating nutrition into oncology care.^12^ CRT is a major treatment modality for early NPC (stage II–IVa), and concurrent CRT has exhibited definite efficacy.^2^^,^^10^ However, the incidence rates of skin, mucous membrane, and digestive tract reactions caused by chemoradiation and acute toxicity reactions are relatively high, which degrade the nutritional status and quality of life of patients. The incidence of nutritional risk or undernutrition in patients with NPC during radiotherapy and chemotherapy has been estimated to be 84%.^3^^,^^4^ The NRS2002 score is a commonly used nutritional risk screening tool during otolaryngology diagnosis and treatment. Nutritional intervention and support should be carried out as soon as possible to maintain and improve nutritional status.^13^^,^^14^ However, a single department or specialty cannot accurately diagnose and formulate the best treatment plan for cancer patients, and the nutritional status of patients having cancer remains poor under the conventional nutrition management mode. However, nutrition management has received extensive clinical attention with the development of multidisciplinary teams. Physicians comprehensively evaluate each patient's tolerance for surgery and physical condition, identify difficult cases, and formulate individualized diagnosis and treatment plans to improve treatment efficacy and long-term quality of life. No study has investigated the impacts of multidisciplinary team nutrition management in patients with NPC undergoing CRT, which hinders the wide application of this model.

Based on a multidisciplinary team, a patient-centered concept and the expert opinions of various departments, such as the departments of head and neck oncology, otorhinolaryngology, psychiatry, and rehabilitation and nutrition, we formulated a comprehensive and innovative nutrition management scheme for patients. The results showed that the nutritional indicators in the multidisciplinary team nutrition management group were better than those in the conventional nutrition management group after radiotherapy or CRT, and the indicators for both groups were better than the results of several previous studies.^15^^,^^16^ This is likely related to differences in the interventional programs created by different scholars. Radiation and chemotherapy drugs have serious impacts on hematopoietic and immune functions, thereby gradually increasing the nutritional risk ratio and decreasing nutritional indicators and immune function. A low nutritional status increases the incidence of complications such as infections, which affect disease treatment options and prognosis.^5^, ^6^, ^7^ According to Huang et al,^17^ nutrition management helps to identify patients with low nutritional status in time to provide better nutritional support. With multidisciplinary team nutrition management, each patient's physical condition is comprehensively assessed from multiple angles, improving treatment efficacy. In this study, the acute toxicity level in the two groups increased significantly with accumulated radiation therapy, and mucositis and ulcers contributed to difficulties in eating, which were the main factors associated with decreased treatment compliance and poor treatment outcomes. The acute toxicity level in the experimental group was significantly lower than that in the routine group, indicating that the patients' acute adverse reaction rate under the multidisciplinary team approach was low and that the side effects of radiotherapy were reduced. Multidisciplinary team nutrition management may help patients through this critical period. By the end of the treatment period, the observation indices were significantly different between the two groups. The bone marrow reserve, mucosa, salivary glands, and gastrointestinal functions were affected to different degrees. Nutritional interventions during treatment help reduce acute radiation reactions and relieve the patients' subjective symptoms. Different malnutrition factors independently decrease the survival rate of cancer patients, whereas diet reduction, the NRS2002 score, and concurrent chemotherapy affected nutritional status, indicating the importance of tumor nutrition during treatment.^17^, ^18^, ^19^ Due to individual differences, nutrition management requires the joint efforts of oncologists, nutritionists, and pharmacists. Nutritional risks and malnutrition are detected early with the implementation of the nutrition management model, and standardized and scientific nutritional interventions can be carried out in time, thus effectively improving patient nutritional status and prognosis.

Our research has some limitations. Retrospective research will lead to inevitable selectivity bias. Retrospective research will lead to inevitable selectivity bias, although there was no significant difference in nutritional indicators between the two groups before CRT. However, we should be aware that different economic conditions, living conditions and other factors may affect the nutritional outcome of patients, so a prospective randomized clinical trial is necessary.

## Conclusions

In summary, with nutrition management using a multidisciplinary team approach, the nutritional status of patients can be comprehensively assessed and timely intervention and nutritional support provided which improves the nutritional status of patients during CRT and reduces the incidence of side effects. However, the incidence of long-term complications and the survival of patients after discharge have not been followed up. More rigorous, multi-center, large-sample, and double-blind studies are still needed.

## Data Availability

The data for the results of this study are available at the authors' request. Data are not made public due to privacy and ethical restrictions.

## CRedit author statement

Xueqing Ou: Conceptualization, Methodology, Data curation, Formal analysis, Writing. Hui Chen: Methodology, Writing – Original draft preparation. Ting Qiu: Formal analysis, Data curation. Yajun Yuan: Conceptualization, Methodology, Data collection, Original and Revised draft preparation. Xiaohua Gong: Conceptualization, Methodology, Data curation, Formal analysis, Writing. All authors had full access to all the data in the study, and the corresponding author had final responsibility for the decision to submit for publication. The corresponding author attests that all listed authors meet authorship criteria and that no others meeting the criteria have been omitted.
